# Recent Patents of Interest to Dentists

**Published:** 1907-09

**Authors:** 


### PATENTS.

### RECENT PATENTS OF INTEREST TO DENTISTS.

861356. Mouth Wedge, Robert Buchfeld, Elberfeld, Germany.
861270. Pneumatic Dental Cement Injector, Henry L. Cruttenden, Northfield, Minn.
861874. Dentimeter, Reuben H. Macy, West Palmbeach, Fla.
861598. Waxless Flask, Isidor S. Moscovitz, New York, N. Y.
862694. Dental Separator, Rufus L. Anderson, Plant City, Fla.
862588. Dental Pliers, Lloyd E. Rowley, Jewell, Kans.
862780. Dentists' Cabinet, Wm. H. Woods, Jasper, Mo.
862881. Dental Appliance, Calvin S. Case, Chicago, Ill.
863527. Tooth Fastener, Frank Fritz, Alexandria, Minn.
863006. Handpiece for Dental Engines, Jeffrey H. Springle, Montreal, Quebec, Can.
863478. Dental Engine, Wallace W. Williamson, Syracuse, N. Y.
860840. Tooth Brush, Gustave Strassburger, New York, N. Y.

Copies of above patents may be obtained for fifteen cents each by addressing John A. Saul, Solicitor of Patents, Fendall Building, Washington, D. C.

